# Supplementary material for: Neutrophil to Lymphocyte Ratio in Oropharyngeal Squamous Cell Carcinoma: A Systematic Review and Meta-Analysis
Source: Cancers (Basel). 2023 Jan 28;15(3):802. doi: 10.3390/cancers15030802 (PMC9913392; doi:10.3390/cancers15030802)
Supplement: Supplementary file 1 [file cancers-15-00802-s001.zip › cancers-2087654-supplementary.pdf]

Supplementary Materials: Neutrophil to Lymphocyte Ratio in Oropharyngeal Squamous Cell Carcinoma: A Systematic Review and Meta-Analysis

Juan P. Rodrigo, Mario Sánchez-Canteli, Asterios Triantafyllou, Remco de Bree, Antti A. Mäkitie, Alessandro Franchi, Henrik Hellquist, Nabil F. Saba, Göran Stenman, Robert P. Takes, Cristina Valero, Nina Zidar and Alfio Ferlito

Table S1. Search strategy in PubMed.

Search:
(neutrophil lymphocyte ratio AND ((((head and neck squamous cell carcinoma) OR (head andneck cancer) OR (oropharyngeal squamous cell carcinoma) OR (oropharynx cancer)))) Sort by: Most Recent
("neutrophil s"[All Fields] OR "neutrophiles"[All Fields] OR "neutrophilic"[All Fields] OR "neutrophillic"[AllFields] OR "neutrophils"[MeSH Terms] OR "neutrophils"[All Fields] OR "neutrophil"[All Fields] OR "neutrophile"[All Fields]) AND ("lymphocytes"[MeSH Terms] OR "lymphocytes"[All Fields] OR "lymphocyte count"[MeSH Terms] OR ("lymphocyte"[All Fields] AND "count"[All Fields]) OR "lymphocyte count"[All Fields] OR "lymphocyte"[All Fields] OR "lymphocytic"[All Fields] OR "lymphocyts"[All Fields]) AND ("ratio"[All Fields] OR "ratio s"[All Fields] OR "ratioes"[All Fields] OR "ratios"[All Fields]) AND ("squamous cell carcinoma of head and neck"[MeSH Terms] OR ("squamous"[All Fields] AND "cell"[All Fields] AND "carcinoma"[All Fields] AND "head"[All Fields] AND "neck"[All Fields]) OR "squamous cell carcinoma of head and neck"[All Fields] OR ("head"[All Fields] AND "neck"[All Fields] AND "squamous"[All Fields] AND "cell"[All Fields] AND "carcinoma"[All Fields]) OR "head and neck squamous cell carcinoma"[All Fields] OR ("head and neck neoplasms"[MeSH Terms] OR ("head"[All Fields] AND "neck"[All Fields] AND "neoplasms"[All Fields]) OR "head and neck neoplasms"[All Fields] OR ("head"[All Fields] AND "neck"[All Fields] AND "cancer"[All Fields]) OR "head and neck cancer"[All Fields]) OR ("squamous cell carcinoma of head and neck"[MeSH Terms] OR ("squamous"[All Fields] AND "cell"[All Fields] AND "carcinoma"[All Fields] AND "head"[All Fields] AND "neck"[All Fields]) OR "squamous cell carcinoma of head and neck"[All Fields] OR ("oropharyngeal"[All Fields] AND "squamous"[All Fields] AND "cell"[All Fields] AND "carcinoma"[All Fields]) OR "oropharyngeal squamous cell carcinoma"[All Fields]) OR ("oropharyngeal neoplasms"[MeSH Terms] OR ("oropharyngeal"[All Fields] AND "neoplasms"[All Fields]) OR "oropharyngeal neoplasms"[All Fields] OR ("oropharynx"[All Fields] AND "cancer"[All Fields]) OR "oropharynx cancer"[All Fields]))
Translations
neutrophil: "neutrophil's"[All Fields] OR "neutrophiles"[All Fields] OR "neutrophilic"[All Fields] OR "neutrophillic"[All Fields] OR "neutrophils"[MeSH Terms] OR "neutrophils"[All Fields] OR "neutrophil"[AllFields] OR "neutrophile"[All Fields]
lymphocyte: "lymphocytes"[MeSH Terms] OR "lymphocytes"[All Fields] OR "lymphocyte count"[MeSHTerms] OR ("lymphocyte"[All Fields] AND "count"[All Fields]) OR "lymphocyte count"[All Fields] OR "lymphocyte"[All Fields] OR "lymphocytic"[All Fields] OR "lymphocyts"[All Fields]
ratio: "ratio"[All Fields] OR "ratio's"[All Fields] OR "ratioes"[All Fields] OR "ratios"[All Fields]
head and neck squamous cell carcinoma: "squamous cell carcinoma of head and neck"[MeSH Terms] OR ("squamous"[All Fields] AND "cell"[All Fields] AND "carcinoma"[All Fields] AND "head"[AllFields] AND "neck"[All Fields]) OR "squamous cell carcinoma of head and neck"[All Fields] OR ("head"[All Fields] AND "neck"[All Fields] AND "squamous"[All Fields] AND "cell"[All Fields] AND "carcinoma"[All Fields]) OR "head and neck squamous cell carcinoma"[All Fields]
head and neck cancer: "head and neck neoplasms"[MeSH Terms] OR ("head"[All Fields] AND "neck"[All Fields] AND "neoplasms"[All Fields]) OR "head and neck neoplasms"[All Fields] OR ("head"[All Fields] AND "neck"[All Fields] AND "cancer"[All Fields]) OR "head and neck cancer"[AllFields]
oropharyngeal squamous cell carcinoma: "squamous cell carcinoma of head and neck"[MeSH Terms] OR ("squamous"[All Fields] AND "cell"[All Fields] AND "carcinoma"[All Fields] AND "head"[AllFields] AND "neck"[All Fields]) OR "squamous cell carcinoma of head and neck"[All Fields] OR ("oropharyngeal"[All Fields] AND "squamous"[All Fields] AND "cell"[All Fields] AND "carcinoma"[All Fields]) OR "oropharyngeal squamous cell carcinoma"[All Fields]
oropharynx cancer: "oropharyngeal neoplasms"[MeSH Terms] OR ("oropharyngeal"[All Fields] AND"neoplasms"[All Fields]) OR "oropharyngeal neoplasms"[All Fields] OR ("oropharynx"[All Fields] AND "cancer"[All Fields]) OR "oropharynx cancer"[All Fields]
Results
n = 492

Table S2. The Newcastle-Ottawa Scale (NOS) for assessing the quality of studies in meta-analyses.

References	Selection				Comparability	Exposure			Total Quality score
	Representa-tiveness of the exposed cohort	Selection of the non-ex-posed cohort	Ascertain-ment of ex-posure	Demonstration that outcome of in test was not present at the start of study	Comparability of cohorts on the basis of the design or analysis	Assessment of outcome	Was follow-up long enough for outcomes to occur	Adequacy of follow-up of cohorts	
Ferrandino et al. [13]	1	1	1	1	2	1	1	1	9
So et al. [14]	1	1	1	1	1	1	1	1	8
De Feliceet al. [15]	1	1	1	1	2	1	1	1	9
Lin et al. [16]	1	1	1	1	2	1	0	1	8
Kreinbrink et al. [17]	1	1	1	1	2	1	0	1	8
Lin et al. [18]	1	1	1	1	2	1	1	1	9
Cho et al. [19]	1	1	1	1	2	1	1	1	9
Brewczyński et al. [20]	1	1	1	1	2	1	1	1	9
Staniewska et al. [21]	1	1	1	1	1	1	0	1	7
Young et al. [22]	1	1	1	1	1	1	0	1	7
Charles et al. [23]	1	1	1	1	2	1	0	1	8
Panje et al. [24]	1	1	1	1	2	1	1	1	9
Gorphe et al. [25]	1	1	1	1	1	1	0	1	7
Ng et al. [26]	1	1	1	1	2	1	1	1	9

Table S3. Key findings of the selected studies.

References	Key Findings
Ferrandino et al. [13]	Elevated NLR in HNSCC confers a poor prognosis.
So et al. [14]	Elevated pretreatment NLR was associated with poor DFS in patients with HPV +OPC
De Feliceet al. [15]	N/L could play an important role in non-HPV-related oropharyngeal cancerprogression and indicate prognosis.
Lin et al. [16]	Lymphopenia and NLR as early as 3 months after treatment start may serve asbiomarkers of clinical outcomes in patients with HPV-negative OPC.
Kreinbrink et al. [17]	In a cohort of HPV + OPSCC patients, increasing absolute lymphocyte count isassociated with improved OS and PFS
Lin et al. [18]	In exploratory analyses, elevated post-treatment NLR was associated with riskfor distant metastases and death
Cho et al. [19]	Head and neck cancer tends to be more aggressive in patients with a high NLR,leading to a poorer outcome after radiotherapy.
Brewczyński et al. [20]	A higher pre-treatment NLR and SII were significant poor prognostic factors forboth OS and DFS in the HPV+ group. These associations were not apparent in HPV− patients.
Staniewska et al. [21]	In the study, only NLR proved to be an independent predictor of OS. However,its clinical value is limited due to the relatively low sensitivity and specificity.
Young et al. [22]	The NLR is an independent prognostic factor for locoregional control inoropharyngeal cancer treated with chemoradiotherapy.
Charles et al. [23]	The results show patients with NLR > 5 predict for shorter overall survival
Panje et al. [24]	The NLR remains an independent prognostic factor for patients with OCundergoing radiotherapy independent of the tumor volume.
Gorphe et al. [25]	We found hemoglobin level and the NRL to be independent prognostic factorsin p16-positive OPSCC patients.
Ng et al. [26]	Pre-radiotherapy NLR is an independent prognostic factor in patients with oropharyngeal cancer regardless of HPV status. Patients with lower NLR hadmore favorable OS and disease control.

NLR: neutrophil-to-lymphocyte ratio; SII: systemic inflammatory index; OS: Overall survival; DFS: Disease-free survival; RFS: Recurrence-free survival; PFS: Progression-free survival.

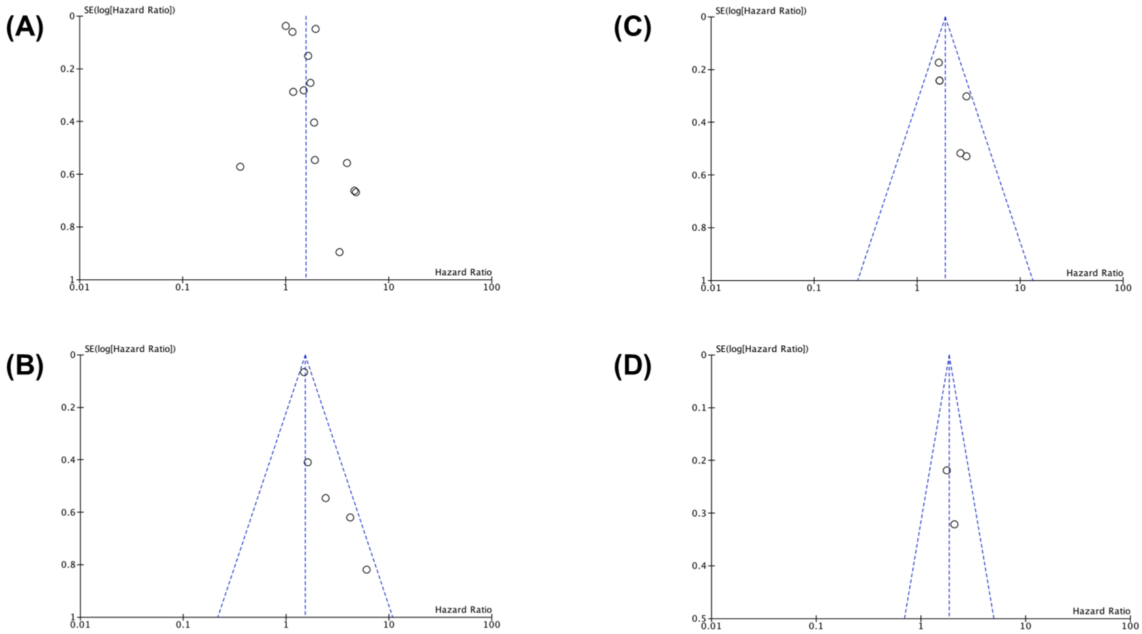

Figure S1. Funnel plots of impact of NLR in "all patients" on overall (**A**), disease-free survival (**B**), recurrence-free survival (**C**) and locoregional control (**D**).

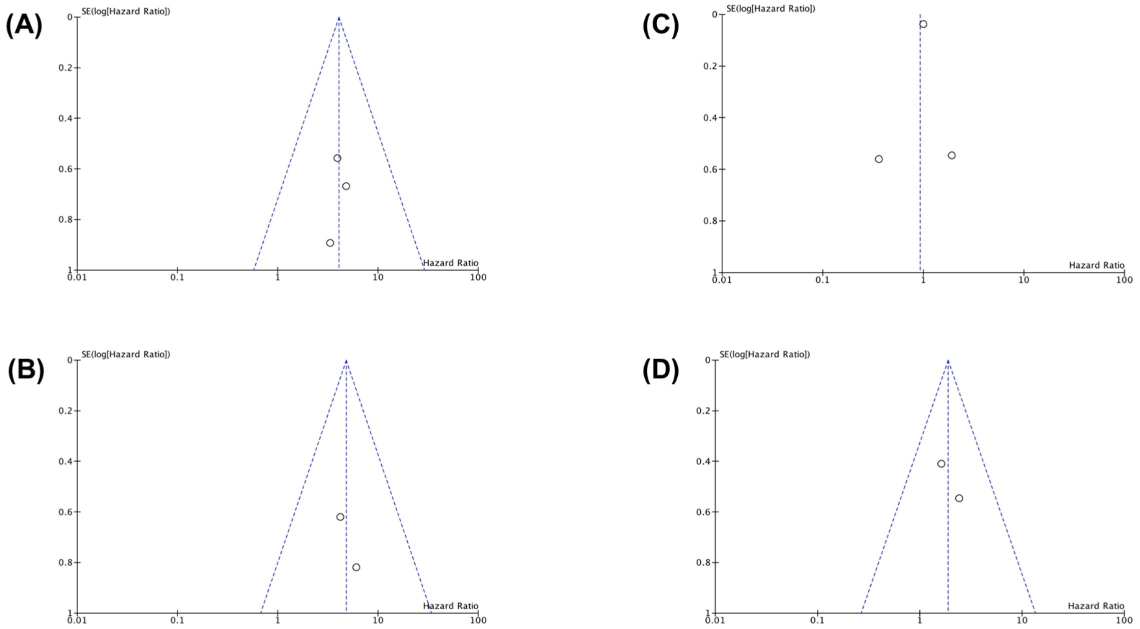

Figure S2. Funnel plots of impact of NLR in "HPV+ patients" on overall survival (**A**) and disease-free survival (**B**) and in "HPV-patients" on overall survival (**C**) and on overall survival (**D**).

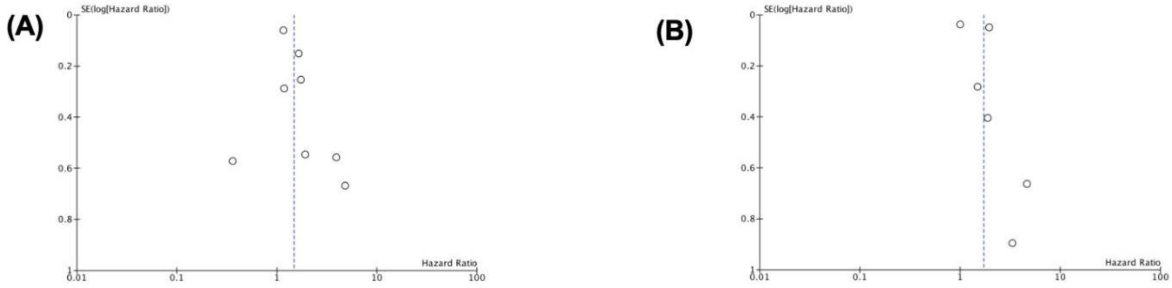

Figure S3. Funnel plots of impact of NLR in “all patients” on overall survival with (**A**) R or CR treatment and (**B**) mixed primary treatment.

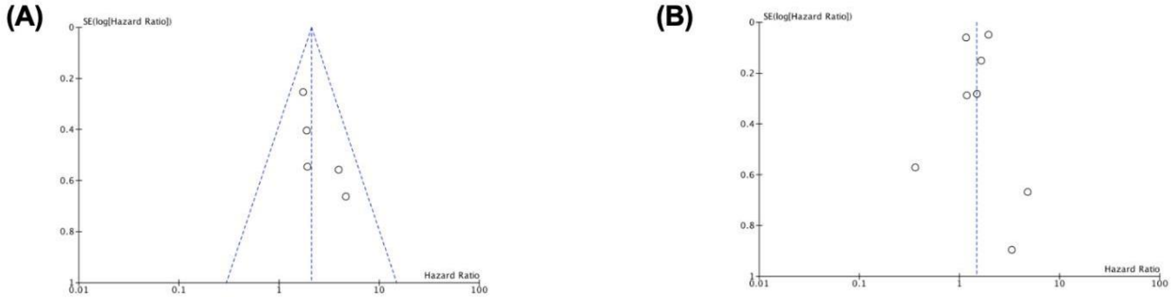

Figure S4. Funnel plots of impact by cut-off value of NLR in “all patients” (**A**) NRL ≥ 3.6 (**B**) < 3.6.
